# Efficacy and tolerability of repetitive transcranial magnetic stimulation for the treatment of obsessive-compulsive disorder in adults: a systematic review and network meta-analysis

**DOI:** 10.1038/s41398-021-01453-0

**Published:** 2021-05-28

**Authors:** Kaili Liang, Hailong Li, Xuan Bu, Xue Li, Lingxiao Cao, Jing Liu, Yingxue Gao, Bin Li, Changjian Qiu, Weijie Bao, Suming Zhang, Xinyu Hu, Haoyang Xing, Qiyong Gong, Xiaoqi Huang

**Affiliations:** 1 Huaxi MR Research Center (HMRRC), Functional and Molecular Imaging Key Laboratory of Sichuan Province, Department of Radiology, West China Hospital of Sichuan University, Chengdu, People’s Republic of China; 2 School of Physical Science and Technology, Sichuan University, Chengdu, People’s Republic of China; 3 Department of Psychiatry, West China Hospital of Sichuan University, Chengdu, People’s Republic of China; 4 Psychoradiology Research Unit of the Chinese Academy of Medical Sciences, West China Hospital of Sichuan University, Chengdu, People’s Republic of China

**Keywords:** Psychiatric disorders, Scientific community

## Abstract

Repetitive transcranial magnetic stimulation (rTMS) has been widely used as an alternative treatment for obsessive-compulsive disorder (OCD). However, the most effective rTMS parameters, such as the targets and stimulation frequencies, remain controversial. Therefore, we aimed to compare and rank the efficacy and tolerability of different rTMS strategies for OCD treatment. We searched five electronic databases from the date of their inception to March 25, 2020. Pairwise meta-analyses and network meta-analyses were performed to synthesize data. We assessed the quality of evidence using the Grading of Recommendations Assessment, Development and Evaluation (GRADE) framework. Twenty-two eligible randomized controlled trials (RCTs) were included. For efficacy, low-frequency (LF) rTMS over the dorsolateral prefrontal cortex (DLPFC; mean difference (MD) 6.34, 95% credible interval (CrI) 2.12–10.42) and supplementary motor area (MD 4.18, 95% CrI 0.83–7.62), and high-frequency rTMS over the DLPFC (MD 3.75, 95% CrI 1.04–6.81) were more effective than sham rTMS. Regarding tolerability, all rTMS treatment strategies were similar to the sham rTMS. The estimated ranking probabilities of treatments showed that LF-rTMS over the DLPFC might be the most effective intervention among all rTMS strategies. However, the quality of evidence regarding efficacy was evaluated as very low. Current evidence suggested a marginal advantage for LF-rTMS over the DLPFC on OCD treatment. High-quality RCTs with low selection and performance bias are needed to further verify the efficacy of specific rTMS strategies for the OCD treatment.

## Introduction

Obsessive-compulsive disorder (OCD), which is characterized by distressing obsessions (repetitive unwanted thoughts or images) or compulsions (repetitive behaviours), is a common and debilitating neuropsychiatric disorder with a lifetime prevalence of 2.5–3% (ref. ^1^). The current first-line treatment strategies for OCD include cognitive–behavioural therapy as a psychological intervention, selective serotonin reuptake inhibitors as a pharmacological intervention or their combination^2^. However, even after receiving standard treatments, approximately half of OCD patients fail to respond well^2,3^, and the proportion is even higher in pragmatic clinical trials^4^, thereby prompting a search for more effective novel treatment strategies, such as repetitive transcranial magnetic stimulation (rTMS), which has been approved by the US Food and Drug Administration (FDA) for the treatment of OCD^5^.

The technique of rTMS is a non-invasive neuromodulatory technique that induces changes in brain activity via a magnetic coil whose field passes through the scalp^6^. As the abnormalities in cortico-striato-thalamo-cortical (CSTC) circuits are hypothesized to be related to symptoms, involving habitual behaviours, anxiety, uncertainty and goal-directed behaviours in OCD patients^3^, and rTMS can modulate brain activity and hence may directly interfere with CSTC loop function^7^, as a result, the abnormalities of brain function in OCD patients could be modulated by the rTMS^7,8^.

Moreover, brain activity changes in different ways in response to different stimulation frequencies. For example, low-frequency (LF) stimulation (usually ≤1 Hz) can temporarily inhibit regional activity, whereas high-frequency (HF) stimulation (usually ≥5 Hz) tends to have an excitatory effect^9^. Therefore, the selection of a treatment target and stimulation frequency plays an important role in the final treatment outcome of OCD patients.

Currently, different combinations of rTMS strategies exist for OCD treatment with no consistent opinion^3,10^. The most common strategies include (1) LF-rTMS or HF-rTMS applied over the dorsolateral prefrontal cortex (DLPFC); (2) LF-rTMS applied over the supplementary motor area (SMA); (3) LF-rTMS applied over the orbitofrontal cortex (OFC); and (4) HF-rTMS applied over the anterior cingulate cortex and medial prefrontal cortex (ACC/mPFC), which uses H-coils to stimulate deeper brain regions in a technique that is also known as deep TMS^11^. Previous pairwise meta-analyses^12–16^ have examined the efficacy of these different strategies of rTMS as a treatment for OCD. However, due to the lack of evidence from direct comparisons of different rTMS treatment strategies in OCD patients, these traditional meta-analysis studies provided limited insight into the overall treatment hierarchy, and thus, consensus regarding which rTMS parameters, such as the stimulation target and frequency, are the most effective is lacking.

Network meta-analyses allow comparisons of efficacy and tolerability among different rTMS treatment strategies, even if the strategies have not been directly compared^17^. Therefore, we performed a network meta-analysis to comprehensively compare and rank the efficacy and tolerability of different rTMS treatment strategies for OCD to obtain a clinically meaningful treatment selection hierarchy.

## Methods

We followed the Preferred Reporting Items for Systematic Reviews and Meta-Analyses (PRISMA) guidelines for network meta-analysis^18^. No protocol or registration details are available.

### Search strategy and selection criteria

We searched the Cochrane Central Register of Controlled Trials, PubMed, Web of Science, Embase and PsycInfo from the date of their inception to March 25, 2020, with no language restrictions. We used the search terms “obsessive compulsive disorder” or “OCD” or “obsessions” or “compulsions” AND “magnetic stimulation” or “rTMS” or “transcranial magnetic”. Two authors (L.K. and L.X.) independently performed the literature search.

We included randomized controlled trials (RCTs) with parallel group or crossover designs involving adults (≥18 years) with a diagnosis of OCD according to the Research Diagnostic Criteria, Diagnostic and Statistical Manual of Mental Disorders or International Classification of Diseases. The studies needed to include the Yale-Brown Obsessive-Compulsive Scale (Y-BOCS) assessment to evaluate the severity of symptoms. We included studies that compared at least two of the following strategies of rTMS: LF-rTMS applied over the DLPFC; HF-rTMS applied over the DLPFC; LF-rTMS applied over the SMA; LF-rTMS applied over the OFC; HF-rTMS applied over the ACC/mPFC; and sham rTMS. Frequencies of 1 Hz or less and 5 Hz or more are defined as LF and HF, respectively. We excluded studies with other study designs, fewer than five subjects with OCD randomized to each study arm, frequencies between 2 and 4 Hz, or fewer than ten treatment sessions.

### Data extraction and outcomes

The data, including the subject characteristics, rTMS parameters and treatment characteristics, were extracted using a standardized data extraction form. The subject characteristics included the mean age, percentage of females and sample size in the active and sham rTMS groups. The rTMS parameters included the location and stimulation frequency. The treatment characteristics included the number of sessions, weeks of treatment and duration of follow-up. We extracted the mean and standard deviation values of the baseline and post-rTMS intervention Y-BOCS scores in both groups, and the number of patients who dropped out due to any adverse events to estimate the drop-out rate. In crossover trials, only data from the first period were extracted to avoid potential carryover effects. In addition, we contacted the first author to request any outcome data that could not be retrieved from the original publication.

### Risk of bias assessment

We initially assessed the risk of bias according to the Cochrane risk of bias tool and classified each study as high risk, unclear risk or low risk^19^. Subsequently, we calculated the percentage of information derived from the studies with low, unclear and high risk of bias in each direct comparison, and then calculated the contributions of the direct comparisons to the effect estimates of the mixed or indirect comparisons, using the methods described by Chaimani et al.^20^.

### Assessment of evidence levels

We evaluated the quality of evidence contributing to each network estimate of the outcomes using the Grading of Recommendations Assessment, Development and Evaluation (GRADE) framework, which characterizes the quality of a body of evidence based on the study limitations, imprecision, heterogeneity and inconsistency, indirectness and publication bias. The GRADE approach enabled us to assign one of four confidence levels (high, moderate, low or very low) to each network estimate of the outcomes^21^.

### Pairwise meta-analysis

Pairwise meta-analyses were conducted to assess each direct comparison within a random-effects model, using the “metan” package in STATA (version 14.0). We calculated the mean difference (MD) in the Y-BOCS score changes and the odds ratio (OR) of the drop-out rates, both with 95% confidence intervals (CIs), and we assessed the statistical heterogeneity in each pairwise comparison with the *I*² statistic value and *P* value.

### Network meta-analysis

We performed random-effects network meta-analyses to assess each direct and indirect comparison within a Bayesian framework using the “gemtc” and “rjags” packages in R (version 3.6.3)^22^. We used the Markov chain Monte Carlo method to obtain the estimated results. Two chains were run simultaneously with different arbitrarily chosen initial values. The convergence of the models was ensured with the Brooks–Gelman–Rubin diagnostic and trace plots. We assumed a common heterogeneity parameter for all treatment comparisons and assessed the global heterogeneity, using the *I*² statistic. The results of all comparisons were summarized as the MD for Y-BOCS score changes and the OR for drop-out rates, both with their 95% credible intervals (CrIs). We estimated the ranking probabilities for each outcome and calculated the surface under the cumulative ranking curve (SUCRA) values to assess the treatment hierarchy^23^.

The assumption of consistency between the direct and indirect evidence was statistically assessed both globally by the test of global inconsistency^20^ and locally by the node-splitting method, which separated the direct evidence from indirect evidence for a particular comparisons^24,25^.

### Small study effects and sensitivity analyses

We used a comparison-adjusted funnel plot, including all direct comparisons to investigate the presence of any dominant publication bias in the network meta-analysis^23^. To evaluate the effect of clinical–demographic characteristics that may be potential effect modifiers for heterogeneous sources, we carried out subgroup analyses using the mean age, percentage of females, number of treatment sessions, the presence of treatment-resistant OCD, the severity of the symptoms based on the Y-BOCS score, the left, right or bilateral location of targets and the subtype of OCD. In addition, we performed sensitivity network meta-analyses for outcomes by (1) excluding studies at high risk of overall bias; (2) excluding studies with long follow-up periods and (3) excluding studies that did not report drop-out data.

## Results

### Characteristics of the included studies

**Fig. 1:**
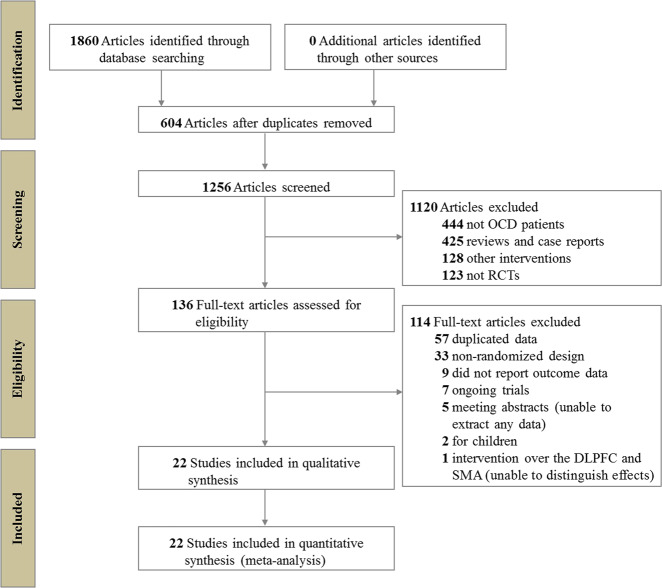
Study selection. OCD obsessive-compulsive disorder, RCTs randomized controlled trials, DLPFC dorsolateral prefrontal cortex, SMA supplementary motor area.

**Fig. 2:**
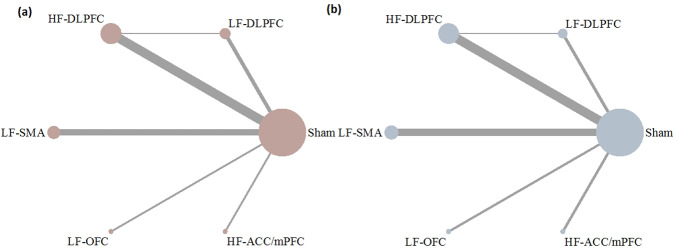
Networks of comparisons for efficacy and tolerability. **a** Network of comparisons for efficacy. **b** Network of comparisons for tolerability. The size of each node is proportional to the total number of randomly assigned participants, and the width of each line is proportional to the number of studies comparing each pair of treatment strategies. LF-DLPFC low-frequency rTMS applied over the dorsolateral prefrontal cortex, HF-DLPFC high-frequency rTMS applied over the dorsolateral prefrontal cortex, LF-SMA low-frequency rTMS applied over the supplementary motor area, LF-OFC low-frequency rTMS applied over the orbitofrontal cortex, HF-ACC/mPFC high-frequency rTMS applied over the anterior cingulate cortex/medial prefrontal cortex.

In total, 1860 references were identified by the search, and the full text of 136 potentially eligible articles was reviewed. We excluded 114 articles for various reasons and included 22 RCTs^11,26–46^ (see Fig. 1). In particular, the study by Kang et al.^47^ was excluded because it targeted both the right DLPFC and the SMA, rendering it impossible to distinguish whether the effect is mediated by the DLPFC, the SMA or the two regions combined. Clinical and demographic characteristics are reported in Table 1. Of a total of 698 patients, 365 were assigned to receive active rTMS and 333 were assigned to receive sham rTMS. A total of 335 (48.0%) of the 698 patients were women, and the mean age was 34.1 years old. The mean number of treatment sessions was 16.5, ranging from 10 to 30. Figure 2 shows the network of direct comparisons for efficacy and tolerability.

**Table 1:** Randomized controlled trials included in the systematic review and network meta-analysis.

Study	Active rTMS			Sham rTMS			TMS parameters			Treatment duration (weeks)	Follow-up (weeks)	Resistant OCD
	*N*	Age	F/M	*N*	Age	F/M	Location	Frequency (Hz)	Sessions			
Alonso et al., 2001	10	39.2 (13.0)	8/2	8	30.3 (9.5)	4/4	R-DLPFC	1 (LF)	18	6	10	No
Prasko et al., 2006	18	28.9 (7.4)	5/13	12	33.4 (8.4)	7/5	L-DLPFC	1 (LF)	10	2	2	Yes
Sachdev et al., 2007	10	29.5 (9.9)	7/3	8	35.8 (8.2)	3/5	L-DLPFC	10 (HF)	10	2	2	Yes
Mantovani et al., 2009	9	39.7 (8.6)	4/5	9	39.4 (10.2)	3/6	B-SMA	1 (LF)	20	4	4	Yes
Ruffini et al., 2009	16	41.5 (NA)	6/10	7	39.3 (NA)	3/4	L-OFC	1 (LF)	15	3	3	Yes
Badawy et al., 2010	20	26.0 (5.7)	8/12	20	28.9 (5.7)	13/7	L-DLPFC	20 (HF)	15	3	3	No
Sarkhel et al., 2010	21	29.4 (6.6)	11/10	21	32.0 (7.8)	8/13	R-DLPFC	10 (HF)	20	4	4	No
Mansur et al., 2011	13	42.1 (11.9)	6/7	14	39.3 (13.9)	8/6	R-DLPFC	10 (HF)	30	6	6	Yes
Gomes et al., 2012	12	35.5 (7.5)	8/4	10	37.5 (6.0)	5/5	B-SMA	1 (LF)	10	2	2	Yes
Ma et al., 2014	25	27.2 (8.9)	8/17	21	29.7 (9.5)	8/13	B-DLPFC	8–12 (HF)	10	2	2	No
Nauczyciel et al., 2014	10	40.0 (NA)	7/2	9	39.0 (NA)	8/2	R-OFC	1 (LF)	10	1	1	Yes
Haghighi et al., 2015	10	34.9 (5.9)	3/7	11	36.6 (4.0)	6/5	B-DLPFC	20 (HF)	20	2	2	Yes
Seo et al., 2016	14	34.6 (9.8)	6/8	13	36.3 (12.5)	7/6	R-DLPFC	1 (LF)	15	3	3	Yes
Elbeh et al., 2016	15	26.8 (5.2)	4/11	15	25.5 (4.0)	5/10	R-DLPFC	1 (LF)	10	2	2	No
	15	28.9 (3.9)	6/9	15	25.5 (4.0)	5/10	R-DLPFC	10 (HF)	10	2	2	No
Hawken et al., 2016	10	33.0 (10.0)	5/5	12	34.0 (14.0)	6/6	B-SMA	1 (LF)	25	6	6	Yes
Jahangard et al., 2016	5	32.4 (9.0)	4/1	5	33.8 (5.8)	3/2	B-DLPFC	20 (HF)	10	2	2	Yes
Pelissolo et al., 2016	20	39.1 (10.4)	13/7	16	42.3 (10.6)	11/8	B-SMA	1 (LF)	20	4	4	Yes
Shayganfard et al., 2016	5	33.8 (9.6)	4/1	5	33.2 (7.9)	2/3	B-DLPFC	20 (HF)	10	2	2	Yes
Carmi et al., 2017	16	36.0 (2.1)	7/9	14	35.0 (3.5)	7/7	ACC/mPFC	20 (HF)	25	5	5	Yes
Arumugham et al., 2018	19	27.7 (7.9)	3/16	17	30.7 (10.4)	5/12	B-SMA	1 (LF)	18	3	3	Yes
Carmi et al., 2019	47	41.1 (12.0)	27/20	47	36.5 (11.4)	28/19	ACC/mPFC	20 (HF)	29	6	6	Yes
Zhang et al., 2019	25	32.2 (13.3)	10/15	24	39.4 (17.0)	10/14	B-SMA	1 (LF)	20	4	4	No

*LF* low frequency, *HF* high frequency, *L-DLPFC* rTMS applied over the left dorsolateral prefrontal cortex, *R-DLPFC* rTMS applied over the right dorsolateral prefrontal cortex, *B-DLPFC* rTMS applied over the bilateral dorsolateral prefrontal cortex, *B-SMA* rTMS applied over the bilateral supplementary motor area, *L-OFC* rTMS applied over the left orbitofrontal cortex, *R-OFC* rTMS applied over the right orbitofrontal cortex, *ACC/mPFC* rTMS applied over the anterior cingulate cortex/medial prefrontal cortex.

### Pairwise meta-analysis

**Fig. 3:**
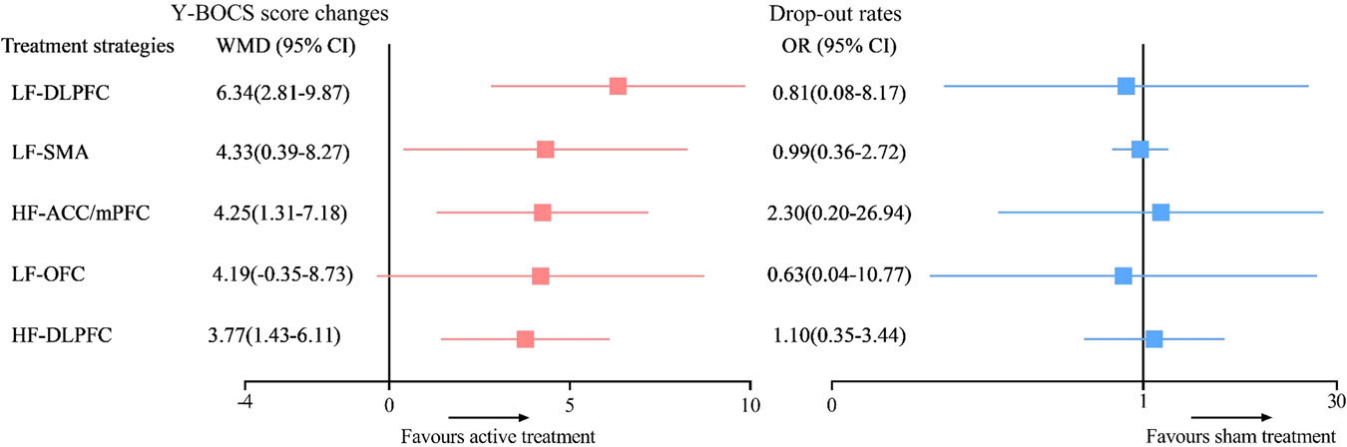
Pairwise meta-analysis of efficacy and tolerability. WMD weighted mean difference, OR odds ratio, CI confidence interval, LF-DLPFC low-frequency rTMS applied over the dorsolateral prefron tal cortex, HF-DLPFC high-frequency rTMS applied over the dorsolateral prefrontal cortex, LF-SMA low-frequency rTMS applied over the supplementary motor area, LF-OFC low-frequency rTMS applied over the orbitofrontal cortex, HF-ACC/mPFC high-frequency rTMS applied over the anterior cingulate cortex/medial prefrontal cortex.

According to the direct evidence (see Fig. 3), LF-rTMS applied over the DLPFC and SMA, and HF-rTMS applied over the DLPFC and ACC/mPFC were significantly more efficacious than sham rTMS in terms of the Y-BOCS score changes (MD (95% CI)): 6.34 (2.81–9.87); 4.33 (0.39–8.27); 3.77 (1.43–6.11); 4.25 (1.31–7.18), respectively), and there was no significant difference in the Y-BOCS score changes between LF-rTMS applied over the OFC and sham rTMS (MD (95% CI)): 4.19 (−0.35 to 8.73)). Regarding tolerability, no significant differences were found in the drop-out rates between active and sham rTMS.

### Network meta-analysis

**Fig. 4:**
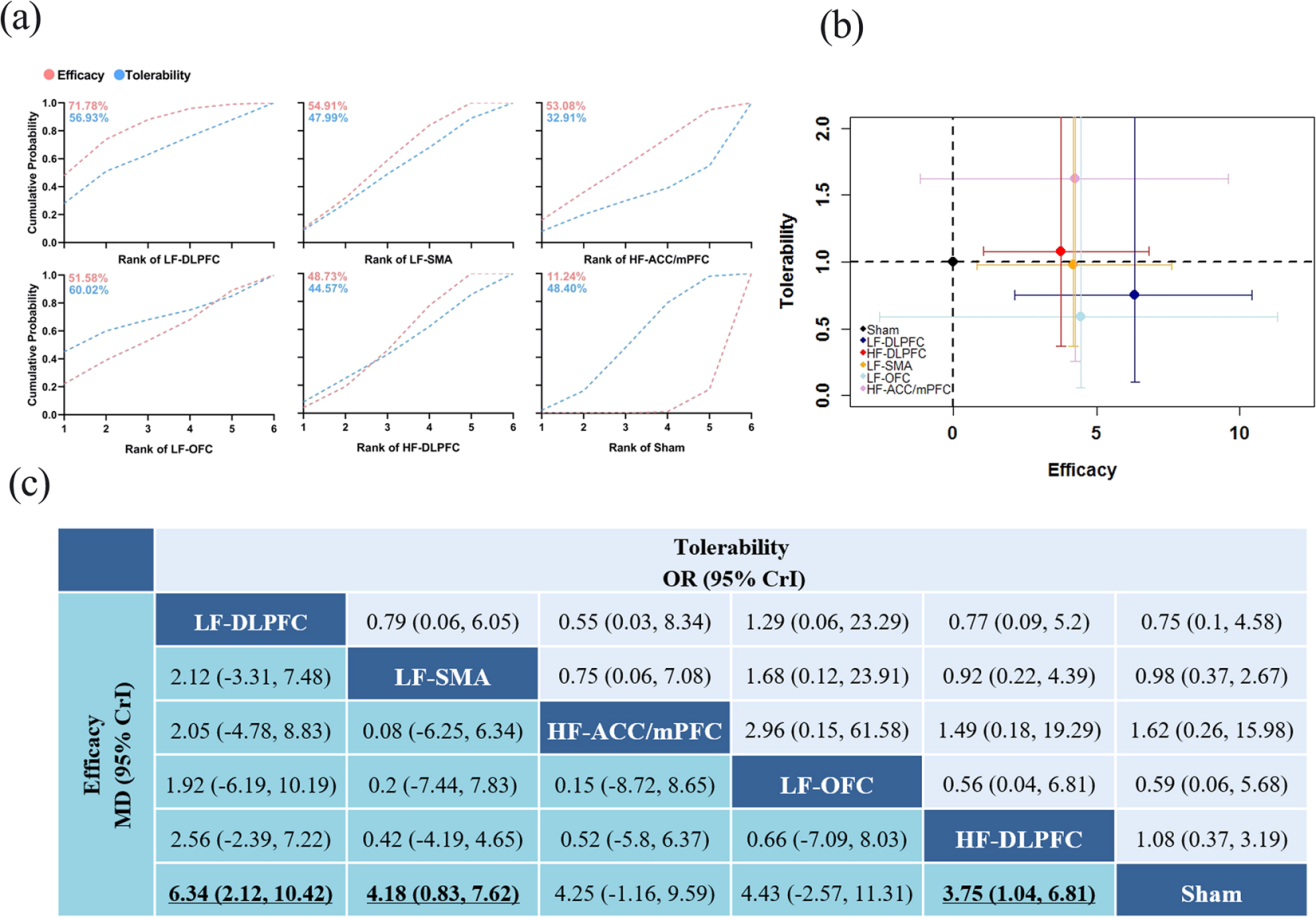
Network meta-analysis of efficacy and tolerability. **a** Ranking regarding efficacy and tolerability are assessed by the surface under the cumulative ranking curve (SUCRA) values. A larger SUCRA denotes a more effective and tolerable treatment strategy. **b** Each treatment strategy is represented by a node of a different colour. The abscissa represents symptom changes as a measure of efficacy (larger values denote more effective treatment strategies), and the ordinate represents drop-out rate as a measure of tolerability (smaller values denote more tolerable treatment strategies). The error bars represent the 95% credible intervals (CrIs). **c** The treatment strategies were ordered according to the surface under the cumulative ranking curve (SUCRA) for efficacy. Results are shown as the mean difference for symptom changes (lower triangle) and odds ratio for drop-out rates (upper triangle) estimated from the network meta-analysis comparing every pair of the six treatment strategies. Comparisons among the six treatment strategies should be read from left to right. For efficacy, an MD larger than 0 favours the intervention in the column. For tolerability, an OR <1 favours the intervention in the row. MD mean difference, OR odds ratio, CrI credible interval, LF-DLPFC low-frequency rTMS applied over the dorsolateral prefrontal cortex, HF-DLPFC high-frequency rTMS applied over the dorsolateral prefrontal cortex, LF-SMA low-frequency rTMS applied over the supplementary motor area, LF-OFC low-frequency rTMS applied over the orbitofrontal cortex, HF-ACC/mPFC high-frequency rTMS applied over the anterior cingulate cortex/medial prefrontal cortex.

The network meta-analysis results are presented in Fig. 4. In terms of efficacy, LF-rTMS applied over the DLPFC (MD 6.34, 95% CrI 2.12–10.42) and SMA (MD 4.18, 95% CrI 0.83–7.62) and HF-rTMS applied over the DLPFC (MD 3.75, 95% CrI 1.04–6.81) were significantly more effective than sham rTMS in terms of Y-BOCS score changes. In terms of tolerability, no significant differences in the drop-out rate were found among the different strategies of rTMS.

The ranking based on cumulative probability plots and the SUCRA value is presented in Fig. 4a. In terms of efficacy, LF-rTMS applied over the DLPFC and SMA ranked first and second, respectively (SUCRA: 71.78 and 54.91%). In terms of tolerability, LF-rTMS applied over the OFC (SUCRA: 60.02%) was the most tolerated, followed by LF-rTMS applied over the DLPFC (SUCRA: 56.93%).

### Heterogeneity and inconsistency analyses

The global *I*² values, 73.5% for efficacy and 0.0% for tolerability, suggested the presence of high heterogeneity in MD for Y-BOCS score changes. Substantial heterogeneity for efficacy was present in HF-rTMS applied over the DLPFC vs sham rTMS (66.4%), LF-rTMS applied over the SMA vs sham rTMS (84.1%) and HF-rTMS applied over the ACC/mPFC vs sham rTMS (63.0%). The test of global inconsistency did not show the presence of statistical inconsistency for efficacy (*P* = 0.442) and tolerability (*P* = 0.987). The test of local inconsistency from the node-splitting model did not show statistical inconsistency for the comparison between LF-rTMS applied over the DLPFC, and HF-rTMS applied over the DLPFC for efficacy (*P* = 0.396) and tolerability (*P* = 0.819).

### Small study effects and sensitivity analyses

**Fig. 5:**
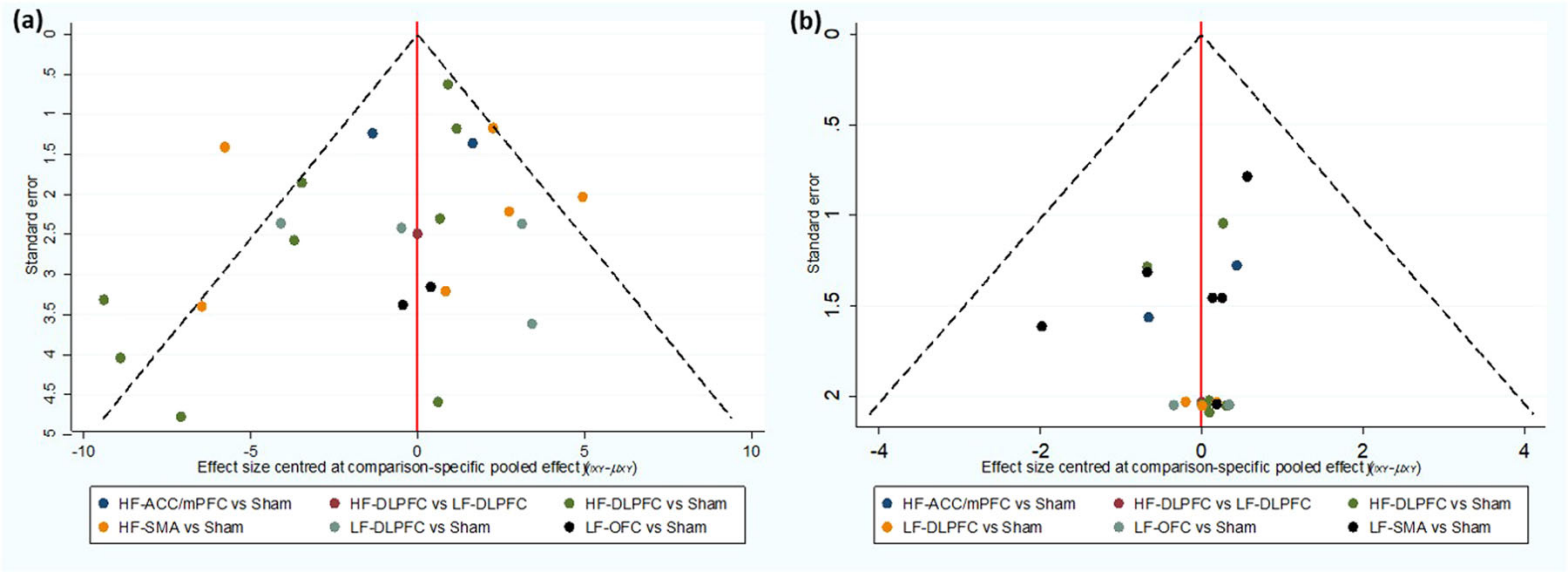
Comparison-adjusted funnel plots for efficacy and tolerability. **a** Comparison-adjusted funnel plot for efficacy. **b** Comparison-adjusted funnel plot for tolerability. LF-DLPFC low-frequency rTMS applied over the dorsolateral prefrontal cortex, HF-DLPFC high-frequency rTMS applied over the dorsolateral prefrontal cortex, LF-SMA low-frequency rTMS applied over the supplementary motor area, LF-OFC low-frequency rTMS applied over the orbitofrontal cortex, HF-ACC/mPFC high-frequency rTMS applied over the anterior cingulate cortex/medial prefrontal cortex.

The comparison-adjusted funnel plots may suggest the presence of asymmetry for efficacy, especially for the comparison between HF-rTMS applied over DLPFC and sham rTMS, and for tolerability, it was not suggestive of the presence of publication bias (see Fig. 5). In the subgroup analyses, we found that the LF-rTMS applied over the DLPFC appeared to be significantly more effective than sham only in the age range of <35 years and in the datasets with smaller percentages of females, and we also found that LF-rTMS applied over the DLPFC was significantly more effective than sham only in the treatment non-resistant OCD patients and in the right side of the brain region (Supplementary Table 1), which may explain the origin of the high heterogeneity for efficacy. We also carried out sensitivity analyses by excluding one study with a high risk of overall bias^32^, excluding one study that had a long follow-up period^26^ and excluding two studies that did not report drop-out data^30,42^; the results of these analyses were not materially different from those of the primary analyses (Supplementary Fig. 1).

### Evaluation of the risk of bias

**Fig. 6:**
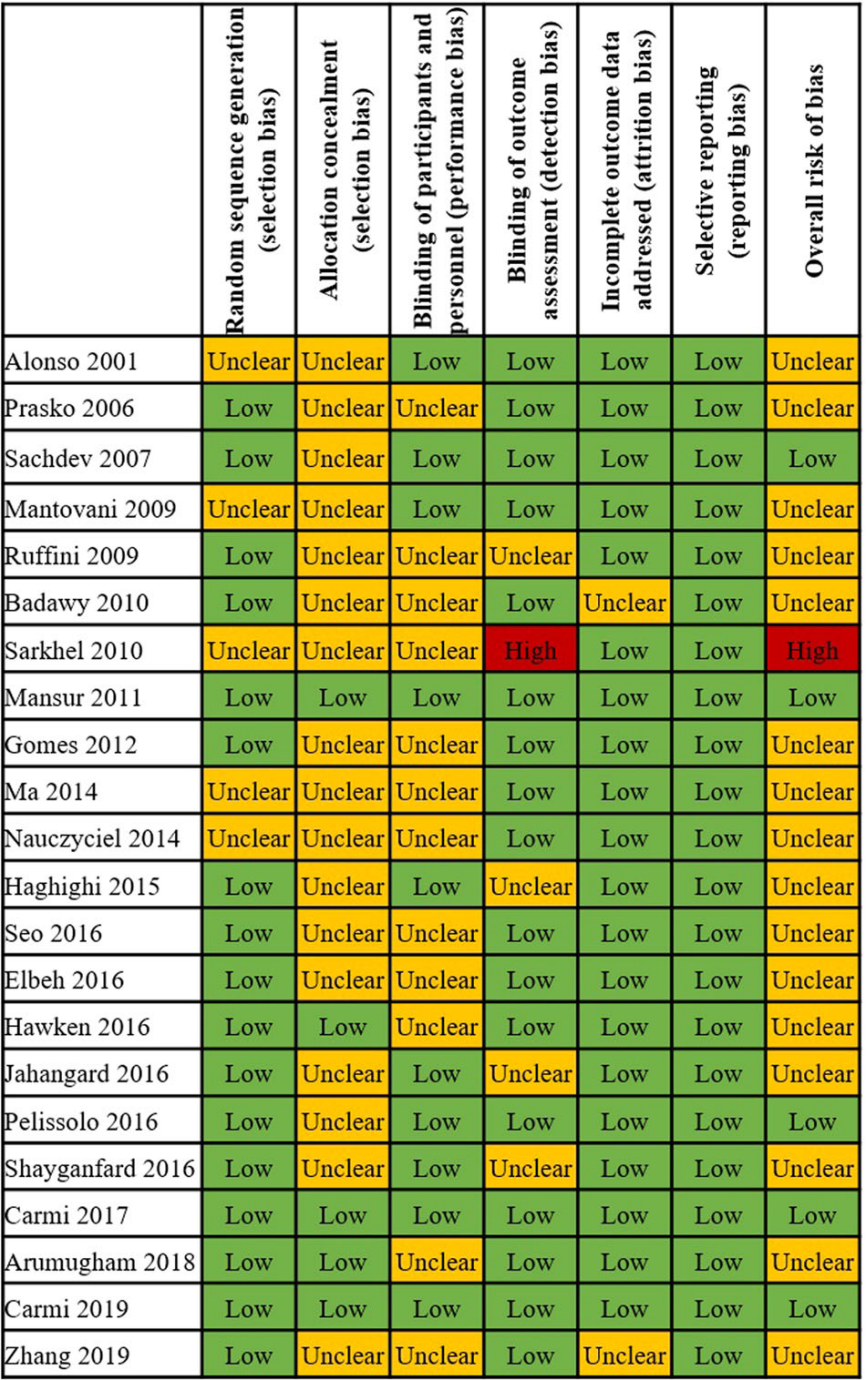
Risk of bias assessment of the included studies. Risk of bias was assessed according to the Cochrane risk of bias tool. Low risk: low risk in all areas or low risk in all areas except for unclear risk in allocation concealment from selection bias. Unclear risk: at least one unclear risk of bias area, except for allocation concealment from selection bias. High risk: at least one high risk of bias in any area.

Regarding the risk of bias, we found that the percentages of studies with low, unclear and high risk of bias were 22.7%, 72.7% and 4.6%, respectively. Most instances of an unclear risk of bias occurred due to allocation concealment in the area of selection bias and blinding of personnel in the area of performance bias (see Fig. 6). The contribution summary of the risk of bias assessment in each direct comparison and each network estimate is shown in Supplementary Figs. 2 and 3, and Supplementary Tables 2 and 3.

### Evaluation of evidence levels

According to the GRADE framework, the quality of the evidence related to the overall ranking of efficacy and tolerability was very low and low, respectively. Regarding efficacy, there was low-quality evidence in the comparisons between LF-rTMS applied over the DLPFC and sham rTMS, and there was very low-quality evidence in the comparisons among HF-rTMS applied over the DLPFC, LF-rTMS applied over the SMA and sham rTMS (Supplementary Table 4). Regarding tolerability, the quality of evidence in the comparison among LF-rTMS applied over the DLPFC, HF-rTMS applied over the DLPFC, LF-rTMS applied over the SMA and sham rTMS was low (Supplementary Table 5).

## Discussion

The network meta-analysis showed that LF-rTMS applied over the DLPFC and SMA, and HF-rTMS applied over the DLPFC were more effective than sham rTMS. Regarding tolerability, all rTMS treatment strategies were similar to sham rTMS. The estimated ranking based on the cumulative probability showed that LF-rTMS applied over the DLPFC might be the most effective intervention among all rTMS strategies for OCD treatment. However, the quality of the evidence of efficacy and tolerability was very low and low, respectively.

The recent evidence-based guidelines for the therapeutic use of rTMS in OCD patients^48^ merely state that LF-rTMS applied over the DLPFC may be efficacious, supporting our findings that LF-rTMS applied over the DLPFC may be the most effective among all rTMS strategies, regarding efficacy rankings. In addition, in the subgroup analyses, we found that LF-rTMS applied over the DLPFC was more effective than sham rTMS only in the right side of the brain region, which was in accordance with the recommendation of the previous evidence-based guideline^48^. These findings may also be explained by abnormalities in the DLPFC linked to deficits in monitoring, working memory and higher-level planning in OCD^49,50^. A previous study^38^ has demonstrated the superiority of LF-rTMS of the right DLPFC, as compared with HF-rTMS or sham rTMS to improve OCD symptoms. This is because OCD may be related to the increased neural activity in prefrontal subcortical circuits, and thus inhibiting the DLPFC area may alleviate OCD related symptoms, such as intrusive thoughts, impulses and higher-level planning, by modulating hyperactivity of CSTC circuit^3,51^.

LF-rTMS applied over the SMA was more effective than sham rTMS and found to be the second most effective treatment. Its efficacy relies on the assumption of deficient inhibitory control over repetitive behaviours in OCD patients associated with hyperactivity in the SMA^52,53^. Previous pairwise meta-analyses^16^ have found that LF-rTMS applied over the SMA yields greater improvements than LF-rTMS applied over the DLPFC for the treatment of OCD patients. This inconsistency may be attributed to the inclusion of two recent studies targeting the SMA^44,46^; therefore, a larger RCT should be performed to further explore the efficacy of this strategy of rTMS.

We did not find that LF-rTMS applied over the OFC or HF-rTMS applied over the ACC/mPFC was more effective than sham rTMS based on currently available evidence. However, the US FDA has approved the use of HF-rTMS applied over the ACC/mPFC for the treatment of OCD patients in adults^5^ based on a large-sample RCT^11^. This rTMS strategy still needs further replication to investigate its reliability.

According to the GRADE framework, the quality of the evidence in the comparison between LF-rTMS applied over the DLPFC and sham rTMS was low, and that in the comparison among HF-rTMS applied over the DLPFC, LF-rTMS applied over the SMA and sham rTMS was very low. We exerted our best effort to include the certainty of evidence of the outcomes to highlight the most robust findings for further clinical interpretation. However, in terms of study limitations based on the GRADE framework, most studies presented an unclear risk of bias due to the allocation concealment in the domain of selection bias and blinding of personnel in the domain of performance bias, which may limit the interpretation of these results. Nonetheless, in the sensitivity analysis, we excluded studies with a high risk of bias, and the results did not substantially change.

In the network meta-analysis, the test of global inconsistency and the local node-splitting method suggested no inconsistency between the direct and indirect evidence. We investigated potentially important clinical and demographical modifiers through subgroup analyses and found that LF-rTMS applied over the DLPFC was more effective than sham rTMS only in those aged <35 years, in the datasets with smaller percentages of females, in the treatment non-resistant OCD patients and in the right side of the brain region, which may explain the sources of high heterogeneity in efficacy. However, without access to individual patient-level data, we cannot be confident regarding the impact of potential modifiers. In addition, since there were only two original studies reporting the symptoms of OCD as moderate and the rest of studies reporting the symptoms of OCD as severe, and there was a lack of the information on the subtype of OCD in the original study, we could not carry out the subgroup analyses to further investigate the effect of the severity of the symptoms and the subtype of OCD. The effects of the severity of the symptoms and the subtype of the OCD need to be further explored in future research.

Finally, in terms of outcomes, we focused on short-term effects; therefore, our conclusions might not apply to the long-term effects of rTMS strategies for the treatment of OCD patients. In the sensitivity analysis, we excluded one trial with a long follow-up period and found that the results were not affected. The long-term effects of rTMS treatments will need to be further investigated in future research.

In summary, we found that LF-rTMS applied over the DLPFC and SMA, and HF-rTMS applied over the DLPFC were more effective than sham rTMS. Regarding tolerability, all rTMS treatment strategies were similar to sham rTMS. Although approved by the US FDA, the application of rTMS for OCD treatment still lacks robust evidence regarding the exact strategy. Recent studies have suggested that rTMS efficacy in neuropsychiatric disorders still needs adequately powered and well-designed RCTs for verification^54–56^. The past three decades have seen the rapid development of neuroimaging techniques and the combined use of MRI and neuronavigation permitting the targeting of individual locations with potential millimetre accuracy^57^. The recently newly established psychoradiological approach^58^ will help provide a more precise target and enhance the provision of personalized management.

## Supplementary information

Supplementary materials

## References

[1] Robbins TW, Vaghi MM, Banca P (2019). Obsessive-compulsive disorder: puzzles and prospects. Neuron.

[2] Hirschtritt ME, Bloch MH, Mathews CA (2017). Obsessive-compulsive disorder: advances in diagnosis and treatment. JAMA.

[3] Stein DJ (2019). Obsessive-compulsive disorder. Nat. Rev. Dis. Prim..

[4] Belotto-Silva C (2012). Group cognitive-behavioral therapy versus selective serotonin reuptake inhibitors for obsessive-compulsive disorder: a practical clinical trial. J. Anxiety Disord..

[5] George MS (2019). Whither TMS: a one-trick pony or the beginning of a neuroscientific revolution?. Am. J. Psychiatry.

[6] Klomjai W, Katz R, Lackmy-Vallée A (2015). Basic principles of transcranial magnetic stimulation (TMS) and repetitive TMS (rTMS). Ann. Phys. Rehabil. Med..

[7] Dougherty DD (2018). Neuroscientifically informed formulation and treatment planning for patients with obsessive-compulsive disorder: a review. JAMA Psychiatry.

[8] Bergfeld, I. O. et al. Invasive and non-invasive neurostimulation for OCD. *Curr. Top. Behav. Neurosci*. 10.1007/7854_2020_206 (2021). 10.1007/7854_2020_206 33550567

[9] Lefaucheur JP (2014). Evidence-based guidelines on the therapeutic use of repetitive transcranial magnetic stimulation (rTMS). Clin. Neurophysiol..

[10] Lusicic A, Schruers KR, Pallanti S, Castle DJ (2018). Transcranial magnetic stimulation in the treatment of obsessive-compulsive disorder: current perspectives. Neuropsychiatr. Dis. Treat..

[11] Carmi L (2019). Efficacy and safety of deep transcranial magnetic stimulation for obsessive-compulsive disorder: a prospective multicenter randomized double-blind placebo-controlled trial. Am. J. Psychiatry.

[12] Berlim MT, Neufeld NH, Van den Eynde F (2013). Repetitive transcranial magnetic stimulation (rTMS) for obsessive-compulsive disorder (OCD): an exploratory meta-analysis of randomized and sham-controlled trials. J. Psychiatr. Res..

[13] Ma ZR, Shi LJ (2014). Repetitive transcranial magnetic stimulation (rTMS) augmentation of selective serotonin reuptake inhibitors (SSRIs) for SSRI-resistant obsessive-compulsive disorder (OCD): a meta-analysis of randomized controlled trials. Int. J. Clin. Exp. Med..

[14] Trevizol AP (2016). Transcranial magnetic stimulation for obsessive-compulsive disorder: an updated systematic review and meta-analysis. J. Ect..

[15] Zhou DD, Wang W, Wang GM, Li DQ, Kuang L (2017). An updated meta-analysis: short-term therapeutic effects of repeated transcranial magnetic stimulation in treating obsessive-compulsive disorder. J. Affect. Disord..

[16] Rehn S, Eslick GD, Brakoulias V (2018). A meta-analysis of the effectiveness of different cortical targets used in repetitive transcranial magnetic stimulation (rTMS) for the treatment of obsessive-compulsive disorder (OCD). Psychiatr. Q..

[17] Mills EJ, Thorlund K, Ioannidis JP (2013). Demystifying trial networks and network meta-analysis. BMJ.

[18] Hutton B (2015). The PRISMA extension statement for reporting of systematic reviews incorporating network meta-analyses of health care interventions: checklist and explanations. Ann. Intern. Med..

[19] Higgins JP (2011). The Cochrane Collaboration’s tool for assessing risk of bias in randomised trials. BMJ.

[20] Chaimani A, Higgins JP, Mavridis D, Spyridonos P, Salanti G (2013). Graphical tools for network meta-analysis in STATA. PLoS ONE.

[21] Salanti G, Del Giovane C, Chaimani A, Caldwell DM, Higgins JP (2014). Evaluating the quality of evidence from a network meta-analysis. PLoS ONE.

[22] Neupane B, Richer D, Bonner AJ, Kibret T, Beyene J (2014). Network meta-analysis using R: a review of currently available automated packages. PLoS ONE.

[23] Salanti G, Ades AE, Ioannidis JP (2011). Graphical methods and numerical summaries for presenting results from multiple-treatment meta-analysis: an overview and tutorial. J. Clin. Epidemiol..

[24] Dias S, Welton NJ, Caldwell DM, Ades AE (2010). Checking consistency in mixed treatment comparison meta-analysis. Stat. Med..

[25] van Valkenhoef G, Dias S, Ades AE, Welton NJ (2016). Automated generation of node-splitting models for assessment of inconsistency in network meta-analysis. Res. Synth. Methods.

[26] Alonso P (2001). Right prefrontal repetitive transcranial magnetic stimulation in obsessive-compulsive disorder: a double-blind, placebo-controlled study. Am. J. Psychiatry.

[27] Prasko J (2006). The effect of repetitive transcranial magnetic stimulation (rTMS) on symptoms in obsessive compulsive disorder. A randomized, double blind, sham controlled study. Neuro Endocrinol. Lett..

[28] Sachdev PS, Loo CK, Mitchell PB, McFarquhar TF, Malhi GS (2007). Repetitive transcranial magnetic stimulation for the treatment of obsessive compulsive disorder: a double-blind controlled investigation. Psychol. Med..

[29] Ruffini C (2009). Augmentation effect of repetitive transcranial magnetic stimulation over the orbitofrontal cortex in drug-resistant obsessive-compulsive disorder patients: a controlled investigation. Prim. Care Companion J. Clin. Psychiatry.

[30] Badawy AA, El Sawy H, Abd El Hay M (2010). Efficacy of repetitive transcranial magnetic stimulation in the management of obsessive compulsive disorder. Egypt J. Neurol. Psychiatr. Neurosurg..

[31] Mantovani A, Simpson HB, Fallon BA, Rossi S, Lisanby SH (2010). Randomized sham-controlled trial of repetitive transcranial magnetic stimulation in treatment-resistant obsessive-compulsive disorder. Int. J. Neuropsychopharmacol..

[32] Sarkhel S, Sinha VK, Praharaj SK (2010). Adjunctive high-frequency right prefrontal repetitive transcranial magnetic stimulation (rTMS) was not effective in obsessive-compulsive disorder but improved secondary depression. J. Anxiety Disord..

[33] Mansur CG (2011). Placebo effect after prefrontal magnetic stimulation in the treatment of resistant obsessive-compulsive disorder: a randomized controlled trial. Int. J. Neuropsychopharmacol..

[34] Gomes PV, Brasil-Neto JP, Allam N, Rodrigues de Souza E (2012). A randomized, double-blind trial of repetitive transcranial magnetic stimulation in obsessive-compulsive disorder with three-month follow-up. J. Neuropsychiatry Clin. Neurosci..

[35] Ma XY, Huang YQ, Liao LW, Jin Y (2014). A randomized double-blinded sham-controlled trial of alpha electroencephalogram-guided transcranial magnetic stimulation for obsessive-compulsive disorder. Chin. Med. J..

[36] Nauczyciel C (2014). Repetitive transcranial magnetic stimulation over the orbitofrontal cortex for obsessive-compulsive disorder: a double-blind, crossover study. Transl. Psychiatry.

[37] Haghighi M (2015). Repetitive Transcranial Magnetic Stimulation (rTMS) improves symptoms and reduces clinical illness in patients suffering from OCD-Results from a single-blind, randomized clinical trial with sham cross-over condition. J. Psychiatr. Res..

[38] Elbeh KAM (2016). Repetitive transcranial magnetic stimulation in the treatment of obsessive-compulsive disorders: Double blind randomized clinical trial. Psychiatry Res..

[39] Hawken ER (2016). Transcranial magnetic stimulation of the supplementary motor area in the treatment of obsessive-compulsive disorder: a multi-site study. Int. J. Mol. Sci..

[40] Jahangard L (2016). Repetitive Transcranial magnetic stimulation improved symptoms of obsessive-compulsive disorder, but also cognitive performance: results from a randomized clinical trial with a cross-over design and sham condition. Neuropsychobiology.

[41] Pelissolo A (2016). Repetitive transcranial magnetic stimulation to supplementary motor area in refractory obsessive-compulsive disorder treatment: a sham-controlled trial. Int. J. Neuropsychopharmacol..

[42] Seo HJ (2016). Adjunctive low-frequency repetitive transcranial magnetic stimulation over the right dorsolateral prefrontal cortex in patients with treatment-resistant obsessive-compulsive disorder: a randomized controlled trial. Clin. Psychopharmacol. Neurosci..

[43] Shayganfard M (2016). Repetitive transcranial magnetic stimulation improved symptoms of obsessive-compulsive disorders but not executive functions: results from a randomized clinical trial with crossover design and sham condition. Neuropsychobiology.

[44] Arumugham SS (2018). Augmentation effect of low-frequency repetitive transcranial magnetic stimulation over presupplementary motor area in obsessive-compulsive disorder a randomized controlled trial. J. Ect..

[45] Carmi L (2018). Clinical and electrophysiological outcomes of deep TMS over the medial prefrontal and anterior cingulate cortices in OCD patients. Brain Stimul..

[46] Zhang K (2019). Impact of serotonin transporter gene on rTMS augmentation of SSRIs for obsessive compulsive disorder. Neuropsychiatr. Dis. Treat..

[47] Kang JI, Kim CH, Namkoong K, Lee CI, Kim SJ (2009). A randomized controlled study of sequentially applied repetitive transcranial magnetic stimulation in obsessive-compulsive disorder. J. Clin. Psychiatry.

[48] Lefaucheur JP (2020). Evidence-based guidelines on the therapeutic use of repetitive transcranial magnetic stimulation (rTMS): An update (2014-2018). Clin. Neurophysiol..

[49] van den Heuvel OA (2005). Frontal-striatal dysfunction during planning in obsessive-compulsive disorder. Arch. Gen. Psychiatry.

[50] Nakao T (2009). Working memory dysfunction in obsessive-compulsive disorder: a neuropsychological and functional MRI study. J. Psychiatr. Res..

[51] Milad MR, Rauch SL (2012). Obsessive-compulsive disorder: beyond segregated cortico-striatal pathways. Trends Cogn. Sci..

[52] Rossi S (2005). Hypofunctioning of sensory gating mechanisms in patients with obsessive-compulsive disorder. Biol. Psychiatry.

[53] Yücel M (2007). Functional and biochemical alterations of the medial frontal cortex in obsessive-compulsive disorder. Arch. Gen. Psychiatry.

[54] Amad A (2019). Excess significance bias in repetitive transcranial magnetic stimulation literature for neuropsychiatric disorders. Psychother. Psychosom..

[55] Brunoni AR (2020). Mixing apples and oranges in assessing outcomes of repetitive transcranial stimulation meta-analyses. Psychother. Psychosom..

[56] Amad A (2020). Reply to the Letter to the Editor: “Mixing apples and oranges in assessing outcomes of repetitive transcranial stimulation meta-analyses”. Psychother. Psychosom..

[57] Luber BM (2017). Using neuroimaging to individualize TMS treatment for depression: toward a new paradigm for imaging-guided intervention. NeuroImage.

[58] Huang X, Gong Q, Sweeney JA, Biswal BB (2019). Progress in psychoradiology, the clinical application of psychiatric neuroimaging. Br. J. Radiol..

