# Supplementary material for: Efficacy and tolerability of repetitive transcranial magnetic stimulation for the treatment of obsessive-compulsive disorder in adults: a systematic review and network meta-analysis
Source: Transl Psychiatry. 2021 May 28;11:332. doi: 10.1038/s41398-021-01453-0 (PMC8163761; doi:10.1038/s41398-021-01453-0)
Supplement: Supplementary file 1 — Supplementary materials [file 41398_2021_1453_MOESM1_ESM.docx]

**Supplementary materials**

**Fig. 1 Sensitivity analysis.** Results are mean difference for symptom changes (lower triangle) and odds ratio for drop-out rates (upper triangle) estimated from the network meta-analysis comparing each pair of the 6 interventions with respect to efficacy and tolerability. The treatments were ordered according to SUCRA for efficacy. Comparisons between 6 interventions should be read from left to right. For efficacy, an MD larger than 0 favours the intervention in the column. For tolerability, an OR less than 1 favours the intervention in the row. Abbreviations: SUCRA, surface under the cumulative ranking curve; MD, mean difference; OR, odds ratio; CrI, credible interval; LF-DLPFC, low-frequency rTMS applied over the dorsolateral prefrontal cortex; HF-DLPFC, high-frequency rTMS applied over the dorsolateral prefrontal cortex; LF-SMA, low-frequency rTMS applied over the supplementary motor area; LF-OFC, low-frequency rTMS applied over the orbitofrontal cortex; HF-ACC/mPFC, high-frequency rTMS applied over the anterior cingulate cortex/medial prefrontal cortex.

**Fig. 2** (a) Contribution summary of risk-of-bias assessment of every direct comparison for efficacy. (b) Contribution summary of risk-of-bias assessment of every direct comparison for tolerability.

**Fig. 3** (a) Contribution summary of risk-of-bias assessment of each network estimate for efficacy. (b) Contribution summary of risk-of-bias assessment of each network estimate for tolerability.

**Table 1** Subgroup network meta-analyses of Y-BOCS score changes as an index of efficacy and drop-out rate as an index of tolerability.

**Table 2** Contribution matrix for efficacy.

**Table 3** Contribution matrix for tolerability.

**Table 4** Confidence assessment of Y-BOCS score changes by the GRADE system.

**Table 5** Confidence assessment of drop-out rate by the GRADE system

**Fig. 1**


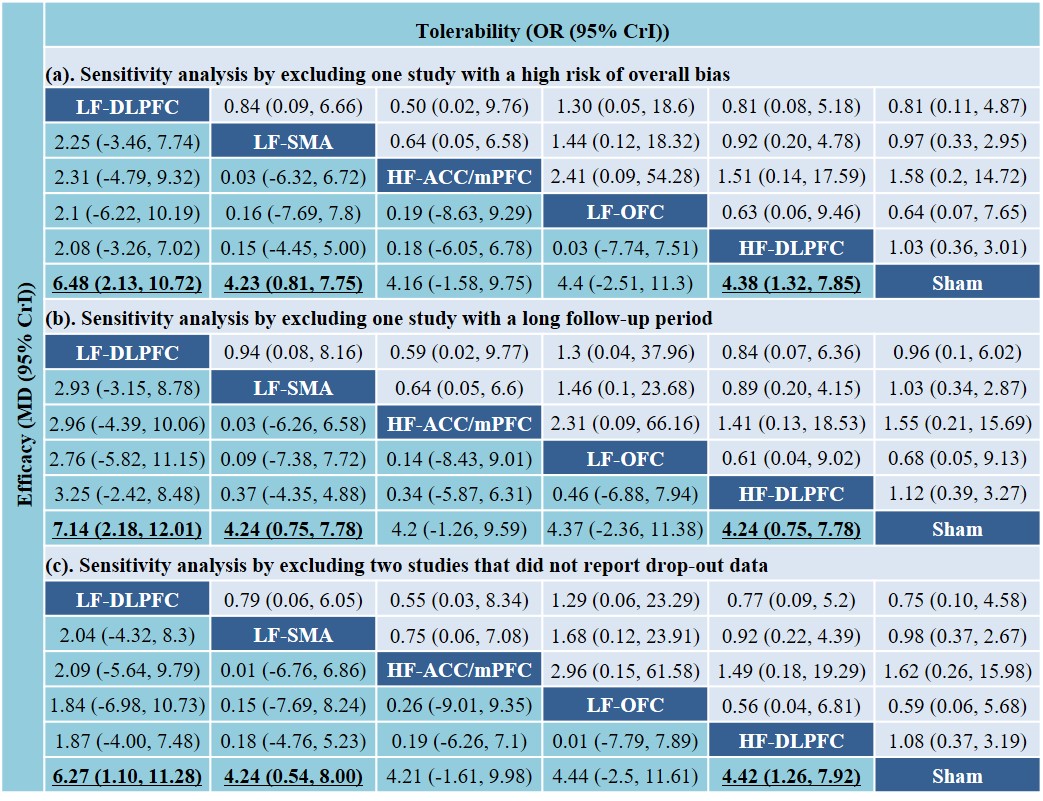


**Fig. 2**

**(a)**

**
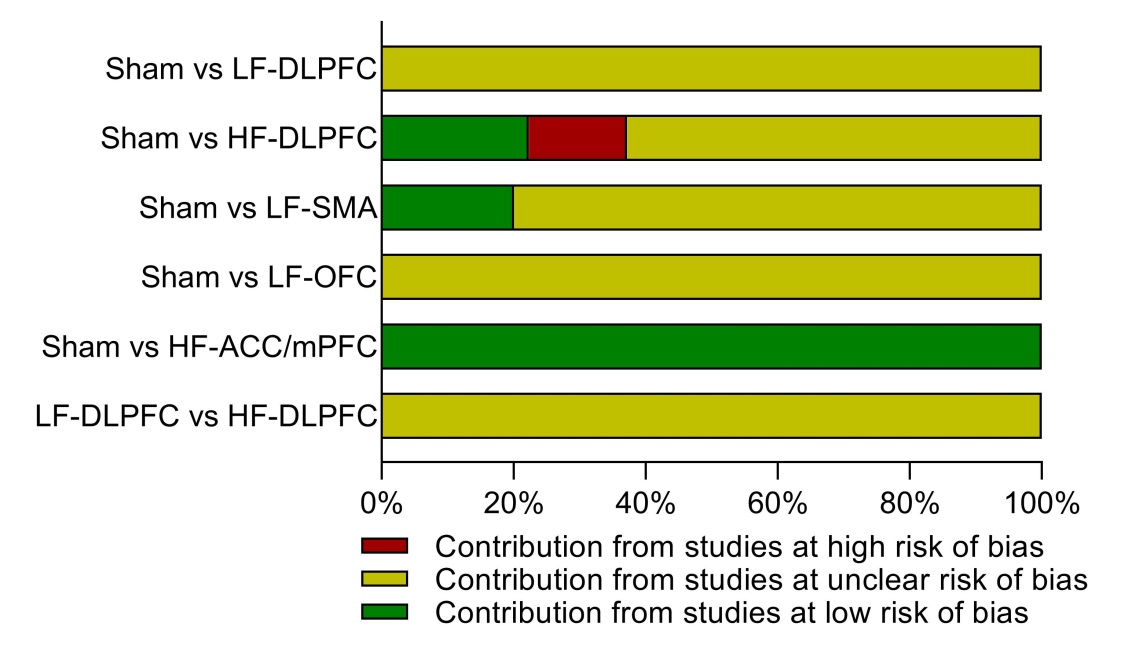
**

**(b)**
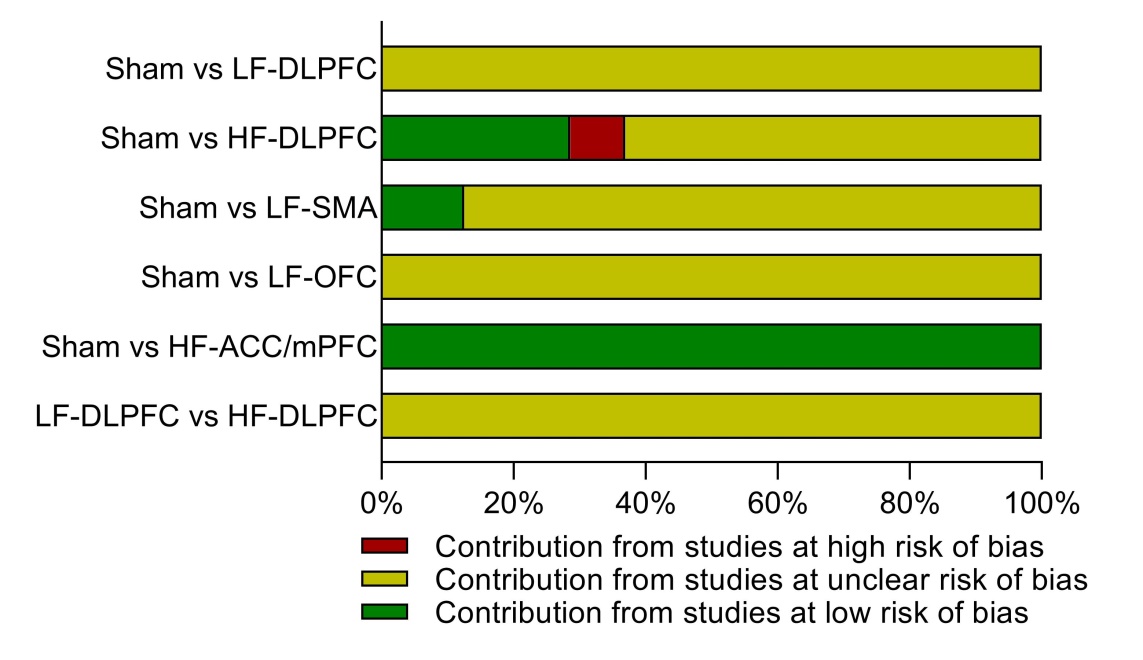


**Fig. 3**


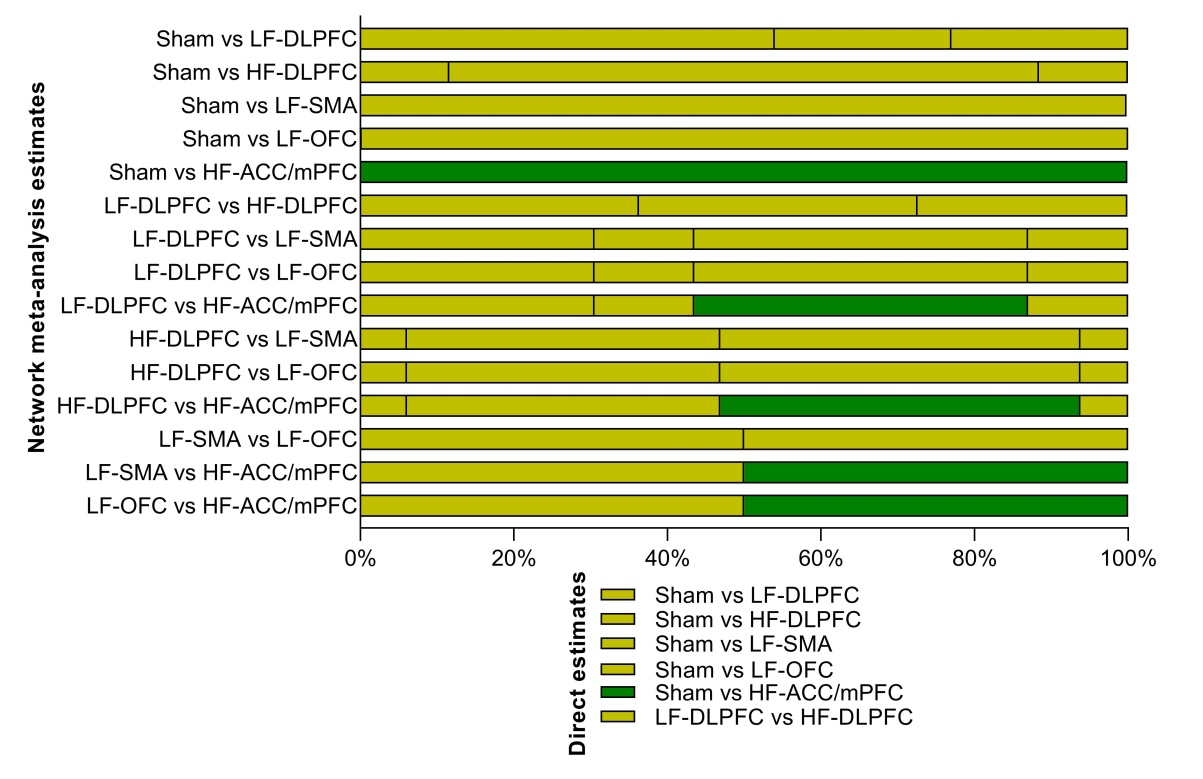
**(a)**

**(b
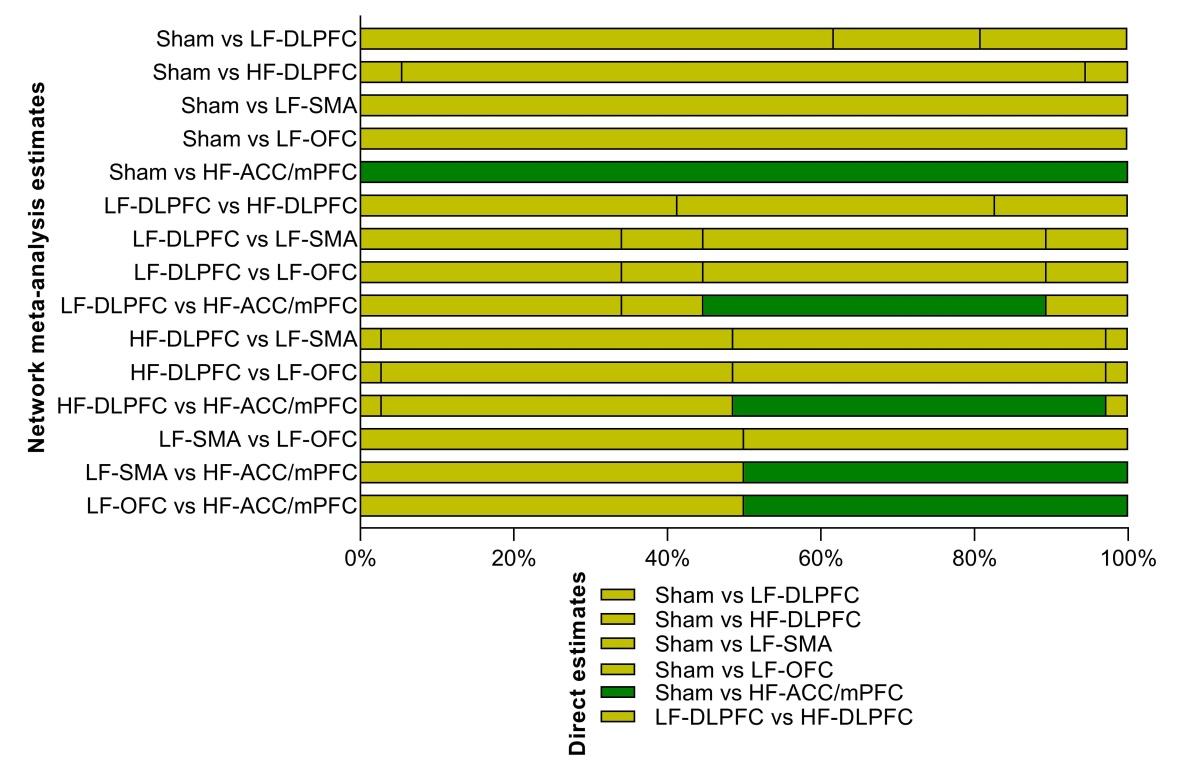
)**

**Table 1 Subgroup network meta-analyses of Y-BOCS score changes as an index of efficacy and drop-out rate as an index of tolerability.**

| **Characteristics** | **Sham vs LF-DLPFC** | **Sham vs HF-DLPFC** | **Sham vs LF-SMA** | **Sham vs LF-OFC** | **Sham vs HF-ACC/mPFC** | **LF-DLPFC vs HF-DLPFC** | **LF-DLPFC vs LF-SMA** | **LF-DLPFC**  **vs LF-OFC** | **LF-DLPFC vs HF-ACC/mPFC** | **HF-DLPFC vs LF-SMA** | **HF-DLPFC vs LF-OFC** | **HF-DLPFC vs HF-ACC/mPFC** | **LF-SMA vs LF-OFC** | **LF-SMA vs HF-ACC/mPFC** | **LF-OFC vs HF-ACC/mPFC** |
| --- | --- | --- | --- | --- | --- | --- | --- | --- | --- | --- | --- | --- | --- | --- | --- |
| **Efficacy** |  |  |  |  |  |  |  |  |  |  |  |  |  |  |  |
| **All studies** | 6.34  (2.12, 10.42) | 3.75  (1.04, 6.81) | 4.18  (0.83, 7.62) | 4.43  (-2.57, 11.31) | 4.25  (-1.16, 9.59) | -2.56  (-7.22, 2.39) | -2.12  (-7.48, 3.31) | -1.92  (-10.19, 6.19) | -2.05  (-8.83, 4.78) | 0.42  (-4.19, 4.65) | 0.66  (-7.09, 8.03) | 0.52  (-5.8, 6.37) | 0.2  (-7.44, 7.83) | 0.08  (-6.25, 6.34) | -0.15  (-8.7, 8.7) |
| **Mean age** |  |  |  |  |  |  |  |  |  |  |  |  |  |  |  |
| < 35 years | 6.91  (0.51, 13.62**)** | 3.37  (-0.26, 7.62) | 5.25  (-2.02, 13.17) | … | … | -3.56  (-10.31, 3.83) | -1.73  (-11.35, 8.3) | … | … | 1.8  (-6.9, 10.3) | … | … | … | … | … |
| ≥ 35 years | 5.28  (-2.82, 13.39) | 5.5  (-2.45, 13.58) | 3.81  (-1.72, 9.08) | 4.36  (-3.86, 12.97) | 4.25  (-3.06, 11.25) | 0.1  (-11.0, 11.67) | -1.58  (-11.23, 8.26) | -1  (-12.4, 11.2) | -1.12  (-11.6, 9.9) | -1.66  (-11.5, 7.8) | -1.09  (-12.79, 10.61) | -1.3  (-12.3, 9.6) | 0.56  (-9.28, 11) | 0.47  (-8.4, 9.4) | -0.17  (-11.4, 10.7) |
| **Female (%)** |  |  |  |  |  |  |  |  |  |  |  |  |  |  |  |
| < 50% | 7.04  (2.41, 11.82) | 3.91  (0.38, 8.67) | 2.13  (-2.48, 6.88) | 3.91  (-5.24, 13.36) | 5.76  (-1.78, 13.63) | -3.12  (-8.35, 3.13) | -4.94  (-11.53, 1.65) | -3.2  (-13.5, 7.4) | -1.23  (-10.23, 7.54) | -1.8  (-8.5, 4) | -0.06  (-10.71, 9.9) | 1.85  (-7.3, 10.0) | 1.8  (-8.5, 12.3) | 3.7  (-5.4, 12.6) | 1.8  (-10.1, 14.2) |
| ≥ 50% | 3.07  (-12.11, 17.39) | 3.72  (-2.27, 10.34) | 4.85  (-4.53, 14.02) | 4.75  (-9.56, 19.07) | 2.67  (-10.12, 15.36) | 0.6  (-14.8, 17.0) | 1.74  (-15.8, 19.06) | 1.6  (-18.0, 22.4) | -0.41  (-19.6, 19.1) | 1.2  (-10.6, 12.1) | 1.0  (-14.6, 16.1) | -1.0  (-15.7, 12.6) | -0.1  (-17.0, 16.9) | -2.2  (-17.9, 13.6) | -1.2  (-21.3, 17.2) |
| **Treatment sessions** |  |  |  |  |  |  |  |  |  |  |  |  |  |  |  |
| < 20 sessions | 6.44  (1.9, 11.15) | 4.08  (0.38, 8.5) | 6.28  (0.12, 11.87) | 4.41  (-2.86, 11.51) | … | -2.39  (-7.94, 3.5) | -0.15  (-7.74, 6.93) | -2.1  (-10.4, 6.5) | … | 2.2  (-5.5, 9.0) | 0.29  (-8.18, 8.34) | … | -1.9  (-10.8, 7.6) | … | … |
| ≥ 20 sessions | … | 3.19  (-2.76, 10.21) | 3.07  (-2.09, 9.31) | … | 4.26  (-3.19, 12.06) | … | … | … | … | -0.06  (-8.9, 8.4) | … | 1.1  (-9.3, 10.6) | … | 1.18  (-8.63, 10.31) | … |
| **Resistant** |  |  |  |  |  |  |  |  |  |  |  |  |  |  |  |
| Yes | 5.18  (-2, 12.3) | 6.18  (1.21, 11.39) | 4.89  (0.38, 9.42) | 4.22  (-3.61, 11.91) | 4.21  (-2.47, 10.87) | -0.99  (-9.93, 7.61) | 0.28  (-8.22, 8.7) | 0.98  (-9.67, 11.51) | 1.03  (-8.97, 10.72) | -1.27  (-8.29, 5.46) | -1.96  (-11.44, 7.11) | -1.99  (-10.4, 6.29) | 0.68  (-8.27, 9.74) | 0.73  (-7.3, 8.64) | -0.05  (-10.28, 10.29) |
| No | 7.24  (0.46, 13.35) | 2.07  (-1.84, 6.75) | 2.05  (-6.44, 10.42) | … | … | 5.13  (-2.61, 11.46) | 5.22  (-5.79, 15.35) | … | … | 0  (-9.81, 9.07) | … | … | … | … | … |
| **Lateralization** |  |  |  |  |  |  |  |  |  |  |  |  |  |  |  |
| Left | 3.35  (-3.06, 9.7) | 0.6  (-3.75, 5.09) | … | 3.99  (-3.75, 11.42) | … | 2.75  (-4.94, 10.51) | … | -0.63  (-10.64, 9.24) | … | … | 3.35  (-5.49, 11.89) | … | … | … | … |
| Right | 7.11  (2.21, 11.72) | 1.44  (-2.57, 6.25) | … | 4.38  (-4.89, 13.72) | … | 5.65  (-0.7, 10.98) | … | 2.7  (-7.87, 13.06) | … | … | 2.88  (-7.58, 12.83) | … | … | … | … |
| Bilateral | … | 8.34  (2.91, 14.06) | 4.3  (0.36, 8.36) | … | 4.25  (-2.29, 10.69) | … | … | … | … | -4.05  (-11.09, 2.72) | … | -4.1  (-12.82, 4.16) | … | 0.05  (-7.52, 7.79) | … |
| **Tolerability** |  |  |  |  |  |  |  |  |  |  |  |  |  |  |  |
| **All studies** | 0.75  (0.1, 4.58) | 1.08  (0.37, 3.19) | 0.98  (0.37, 2.67) | 0.59  (0.06, 5.68) | 1.62  (0.26, 15.98) | 1.44  (0.19, 10.76) | 1.29  (0.18, 13.46) | 0.76  (0.04, 19.87) | 2.24  (0.16, 44.43) | 0.92  (0.22, 4.39) | 0.56  (0.04, 6.81) | 1.49  (0.18, 19.29) | 0.61  (0.05, 6.5) | 1.64  (0.22, 20.28) | 2.96  (0.15, 61.58) |
| **Mean age** |  |  |  |  |  |  |  |  |  |  |  |  |  |  |  |
| < 35 years | 0.77  (0.05, 5.95) | 0.85  (0.29, 2.66) | 1.85  (0.24, 14.79) | … | … | 1.17  (0.14, 15.93) | 2.58  (0.13, 67.11) | … | … | 2.18  (0.19, 22.93) | … | … | … | … | … |
| ≥ 35 years | 0.8  (0.02, 31.91) | 1.63  (0.23, 13.86) | 0.8  (0.24, 2.77) | 0.55  (0.05, 5.87) | 1.59  (0.19, 13.4) | 2.2  (0.03, 132.73) | 1  (0.02, 56.52) | 0.7  (0.01, 59.68) | 2  (0.03, 140.16) | 0.49  (0.04, 5.17) | 0.33  (0.02, 7.94) | 0.91  (0.05, 17.07) | 0.7  (0.05, 10.73) | 2  (0.18, 22.05) | 2.76  (0.12, 72.82) |
| **Female (%)** |  |  |  |  |  |  |  |  |  |  |  |  |  |  |  |
| < 50% | 0.81  (0.09, 6.46) | 0.87  (0.2, 3.66) | 0.86  (0.21, 3.59) | 0.41  (0.01, 13.15) | 0.82  (0.02, 60.32) | 1.09  (0.11, 11.63) | 1.04  (0.08, 12.47) | 0.52  (0.01, 29.84) | 1.01  (0.02, 123.81) | 0.99  (0.13, 6.98) | 0.46  (0.01, 18.42) | 0.93  (0.02, 67.19) | 0.46  (0.01, 21.74) | 0.96  (0.02, 91.15) | 2.03  (0.01, 612.78) |
| ≥ 50% | 0.68  (0.01, 17.32) | 1.19  (0.27, 5.71) | 0.8  (0.09, 8.81) | 0.84  (0.04, 33.13) | 2.19  (0.13, 46.36) | 1.8  (0.04, 100.14) | 1.3  (0.02, 96.34) | 1.37  (0.01, 296.43) | 3.62  (0.05, 383.84) | 0.71  (0.04, 11.18) | 0.73  (0.02, 34.17) | 1.91  (0.08, 56.02) | 1.05  (0.02, 82.37) | 2.68  (0.08, 123.43) | 2.72  (0.02, 191.8) |
| **Treatment sessions** |  |  |  |  |  |  |  |  |  |  |  |  |  |  |  |
| < 20 sessions | 0.75  (0.12, 4.39) | 0.87  (0.23, 3.28) | 0.85  (0.07, 8.12) | 0.53  (0.05, 5.87) | … | 1.14  (0.14, 8.99) | 1.14  (0.05, 20.03) | 0.68  (0.04, 13.96) | … | 0.94  (0.07, 14.15) | 0.61  (0.04, 9.04) | … | 0.66  (0.02, 15.79) | … | … |
| ≥ 20 sessions | … | 1.47  (0.25, 9.04) | 1.05  (0.32, 3.47) | … | 1.4  (0.19, 13.98) | … | … | … | … | 0.72  (0.09, 5.99) | … | 0.96  (0.07, 16.27) | … | 1.34  (0.12, 18.06) | … |
| **Resistant** |  |  |  |  |  |  |  |  |  |  |  |  |  |  |  |
| Yes | 0.65  (0.02, 32.43) | 1.21  (0.3, 5.25) | 1.4  (0.38, 5.42) | 0.57  (0.04, 6.27) | 1.54  (0.2, 14.4) | 0.52  (0.01, 37.61) | 0.47  (0.01, 26.89) | 1.14  (0.01, 154.01) | 0.44  (0.01, 27.95) | 1.15  (0.15, 7.93) | 0.46  (0.02, 7.51) | 1.23  (0.1, 18.95) | 2.41  (0.18, 52.43) | 0.87  (0.07, 11.86) | 2.74  (0.11, 78.77) |
| No | 0.89  (0.1, 6.59) | 0.92  (0.23, 3.82) | 0.54  (0.09, 2.86) | … | … | 0.93  (0.1, 9.85) | 1.65  (0.11, 24.57) | … | … | 0.57  (0.06, 5.22) | … | … | … | … | … |
| **Lateralization** |  |  |  |  |  |  |  |  |  |  |  |  |  |  |  |
| Left | 0.71  (0.02, 27.73) | 0.77  (0.02, 35.36) | … | 0.38  (0.01, 16.97) | … | 0.88  (0.01, 132.29) | … | 1.87  (0.01, 464.07) | … | … | 0.5  (0, 72.98) | … | … | … | … |
| Right | 1.07  (0.12, 10.04) | 1.34  (0.24, 7.56) | … | 0.95  (0.03, 30.74) | … | 0.84  (0.06, 8.86) | … | 1.14  (0.02, 59.66) | … | … | 0.7  (0.01, 30.77) | … | … | … | … |
| Bilateral | … | 0.94  (0.21, 4.04) | 1  (0.34, 2.8) | … | 1.66  (0.21, 13.11) | … | … | … | … | 1.03  (0.18, 6.48) | … | 1.8  (0.14, 22.05) | … | 0.59  (0.06, 5.67) | … |

**Table 2 Contribution matrix for efficacy.**

| **Direct comparison** | **Comparison** | **N** | **Sham vs LF-DLPFC** | **Sham vs HF-DLPFC** | **Sham vs LF-SMA** | **Sham vs LF-OFC** | **Sham vs HF-ACC/mPFC** | **LF-DLPFC vs HF-DLPFC** |
| --- | --- | --- | --- | --- | --- | --- | --- | --- |
|  | Sham vs LF-DLPFC | 4 | 54% | 23% | 0 | 0 | 0 | 23% |
|  | Sham vs HF-DLPFC | 9 | 11.6% | 76.8% | 0 | 0 | 0 | 11.6% |
|  | Sham vs LF-SMA | 6 | 0 | 0 | 100% | 0 | 0 | 0 |
|  | Sham vs LF-OFC | 2 | 0 | 0 | 0 | 100% | 0 | 0 |
|  | Sham vs HF-ACC/mPFC | 2 | 0 | 0 | 0 | 0 | 100% | 0 |
|  | LF-DLPFC vs HF-DLPFC | 1 | 36.3% | 36.3% | 0 | 0 | 0 | 27.4% |
| **Indirect comparison** | LF-DLPFC vs LF-SMA |  | 30.5% | 13% | 43.5% | 0 | 0 | 13% |
|  | LF-DLPFC vs LF-OFC |  | 30.5% | 13% | 0 | 43.5% | 0 | 13% |
|  | LF-DLPFC vs HF-ACC/mPFC |  | 30.5% | 13% | 0 | 0 | 43.5% | 13% |
|  | HF-DLPFC vs LF-SMA |  | 6.1% | 40.8% | 46.9% | 0 | 0 | 6.2% |
|  | HF-DLPFC vs LF-OFC |  | 6.1% | 40.8% | 0 | 46.9% | 0 | 6.2% |
|  | HF-DLPFC vs HF-ACC/mPFC |  | 6.1% | 40.8% | 0 | 0 | 46.9% | 6.2% |
|  | LF-SMA vs LF-OFC |  | 0 | 0 | 50% | 50% | 0 | 0 |
|  | LF-SMA vs HF-ACC/mPFC |  | 0 | 0 | 50% | 0 | 50% | 0 |
|  | LF-OFC vs HF-ACC/mPFC |  | 0 | 0 | 0 | 50% | 50% | 0 |

**Table 3 Contribution matrix for tolerability.**

| **Direct comparison** | **Comparison** | **N** | **Sham vs LF-DLPFC** | **Sham vs HF-DLPFC** | **Sham vs LF-SMA** | **Sham vs LF-OFC** | **Sham vs HF-ACC/mPFC** | **LF-DLPFC vs HF-DLPFC** |
| --- | --- | --- | --- | --- | --- | --- | --- | --- |
|  | Sham vs LF-DLPFC | 3 | 61.8% | 19.1% | 0 | 0 | 0 | 19.1% |
|  | Sham vs HF-DLPFC | 8 | 5.5% | 89% | 0 | 0 | 0 | 5.5% |
|  | Sham vs LF-SMA | 6 | 0 | 0 | 100% | 0 | 0 | 0 |
|  | Sham vs LF-OFC | 2 | 0 | 0 | 0 | 100% | 0 | 0 |
|  | Sham vs HF-ACC/mPFC | 2 | 0 | 0 | 0 | 0 | 100% | 0 |
|  | LF-DLPFC vs HF-DLPFC | 1 | 41.3% | 41.4% | 0 | 0 | 0 | 17.3% |
| **Indirect comparison** | LF-DLPFC vs LF-SMA |  | 34.1% | 10.6% | 44.7% | 0 | 0 | 10.6% |
|  | LF-DLPFC vs LF-OFC |  | 34.1% | 10.6% | 0 | 44.7% | 0 | 10.6% |
|  | LF-DLPFC vs HF-ACC/mPFC |  | 34.1% | 10.6% | 0 | 0 | 44.7% | 10.6% |
|  | HF-DLPFC vs LF-SMA |  | 2.8% | 45.8% | 48.6% | 0 | 0 | 2.8% |
|  | HF-DLPFC vs LF-OFC |  | 2.8% | 45.8% | 0 | 48.6% | 0 | 2.8% |
|  | HF-DLPFC vs HF-ACC/mPFC |  | 2.8% | 45.8% | 0 | 0 | 48.6% | 2.8% |
|  | LF-SMA vs LF-OFC |  | 0 | 0 | 50% | 50% | 0 | 0 |
|  | LF-SMA vs HF-ACC/mPFC |  | 0 | 0 | 50% | 0 | 50% | 0 |
|  | LF-OFC vs HF-ACC/mPFC |  | 0 | 0 | 0 | 50% | 50% | 0 |

**Table 4 Confidence assessment of Y-BOCS score changes by the GRADE system.**

| **Comparison** | **Study limitations** | **Imprecision** | **Heterogeneity and inconsistency** | **Indirectness** | **Publication bias** | **Confidence** |
| --- | --- | --- | --- | --- | --- | --- |
| **Sham**  **vs**  **LF-DLPFC** | 100% of the estimate from studies at unclear risk | MD (95% CrI):  6.34 (2.12 to 10.42) | I^2^ (46.9%) and P value (0.130) in direct comparisons  Only direct comparison and no node-splitting inconsistency | The treatment effects were affected by age and percentage of females in the subgroup analyses | The comparison-adjusted funnel plot for the network did not suggest any dominant publication bias | Low (downgrade by two levels due to study limitations and indirectness) |
| **Sham**  **vs**  **HF-DLPFC** | 100% of the estimate from studies at unclear risk | MD (95% CrI):  3.75 (1.04 to 6.81) | I^2^ (66.4%) and P value (0.002) in direct comparisons  Only direct comparison and no node-splitting inconsistency | The treatment effects were affected by treatment sessions and percentage of females in the subgroup analyses | The comparison-adjusted funnel plot for the network may suggest the presence of publication bias | Very low (downgrade by four levels due to study limitations, heterogeneity, indirectness and publication bias) |
| **Sham**  **vs**  **LF-SMA** | 100% of the estimate from studies at unclear risk | MD (95% CrI):  4.18 (0.83 to 7.62) | I^2^ (84.1%) and P value (< 0.001) in direct comparisons  Only direct comparison and no node-splitting inconsistency | The treatment effects were affected by treatment sessions in the subgroup analyses | The comparison-adjusted funnel plot for the network did not suggest any dominant publication bias | Very low (downgrade by three levels due to study limitations, heterogeneity and indirectness) |
| **Sham**  **vs**  **LF-OFC** | 100% of the estimate from studies at unclear risk | MD (95% CrI):  4.43 (-2.57 to 11.31) | I^2^ (0%) and P value (0.899) in direct comparisons  Only direct comparison and no node-splitting inconsistency | The treatment effects were not affected by clinical modifiers in the subgroup analyses | The comparison-adjusted funnel plot for the network did not suggest any dominant publication bias | Low (downgrade by two levels due to study limitations and imprecision) |
| **Sham**  **vs**  **HF-ACC/mPFC** | 100% of the estimate from studies at low risk | MD (95% CrI):  4.25 (-1.16 to 9.59) | I^2^ (63.0%) and P value (0.100) in direct comparisons  Only direct comparison and no node-splitting inconsistency | The treatment effects were not affected by clinical modifiers in the subgroup analyses | The comparison-adjusted funnel plot for the network did not suggest any dominant publication bias | Moderate (downgrade by one level due to imprecision) |
| **LF-DLPFC**  **vs**  **HF-DLPFC** | 100% of the estimate from studies at unclear risk | MD (95% CrI):  -2.56 (-7.22 to 2.39) | Only one head-to-head study, and no heterogeneity  No inconsistency between the direct and indirect comparison (Node-split p= 0.396) | The treatment effects were not affected by clinical modifiers in the subgroup analyses | The comparison-adjusted funnel plot for the network did not suggest any dominant publication bias | Low (downgrade by two levels due to study limitations and imprecision) |
| **LF-DLPFC**  **vs**  **LF-SMA** | 100% of the estimate from studies at unclear risk | MD (95% CrI):  -2.12 (-7.48 to 3.31) | No head-to-head study and no heterogeneity  Only indirect comparison and no node-splitting inconsistency | The treatment effects were not affected by clinical modifiers in the subgroup analyses | The comparison-adjusted funnel plot for the network did not suggest any dominant publication bias | Low (downgrade by two levels due to study limitations and imprecision) |
| **LF-DLPFC**  **vs**  **LF-OFC** | 100% of the estimate from studies at unclear risk | MD (95% CrI):  -1.92 (-10.19 to 6.19) | No head-to-head study and no heterogeneity  Only indirect comparison and no node-splitting inconsistency | The treatment effects were not affected by clinical modifiers in the subgroup analyses | The comparison-adjusted funnel plot for the network did not suggest any dominant publication bias | Low (downgrade by two levels due to study limitations and imprecision) |
| **LF-DLPFC**  **vs**  **HF-ACC/mPFC** | 56.5% of the estimate from studies at unclear risk, and 43.5% at low risk | MD (95% CrI):  -2.05 (-8.83 to 4.78) | No head-to-head study and no heterogeneity  Only indirect comparison and no node-splitting inconsistency | The treatment effects were not affected by clinical modifiers in the subgroup analyses | The comparison-adjusted funnel plot for the network did not suggest any dominant publication bias | Low (downgrade by two levels due to study limitations and imprecision) |
| **HF-DLPFC**  **vs**  **LF-SMA** | 100% of the estimate from studies at unclear risk | MD (95% CrI):  0.42 (-4.19 to 4.65) | No head-to-head study and no heterogeneity  Only indirect comparison and no node-splitting inconsistency | The treatment effects were not affected by clinical modifiers in the subgroup analyses | The comparison-adjusted funnel plot for the network did not suggest any dominant publication bias | Low (downgrade by two levels due to study limitations and imprecision) |
| **HF-DLPFC**  **vs**  **LF-OFC** | 100% of the estimate from studies at unclear risk | MD (95% CrI):  0.66 (-7.09 to 8.03) | No head-to-head study and no heterogeneity  Only indirect comparison and no node-splitting inconsistency | The treatment effects were not affected by clinical modifiers in the subgroup analyses | The comparison-adjusted funnel plot for the network did not suggest any dominant publication bias | Low (downgrade by two levels due to study limitations and imprecision) |
| **HF-DLPFC**  **vs**  **HF-ACC/mPFC** | 53.1% of the estimate from studies at unclear risk, and 46.9% at low risk | MD (95% CrI):  0.52 (-5.8 to 6.37) | No head-to-head study and no heterogeneity  Only indirect comparison and no node-splitting inconsistency | The treatment effects were not affected by clinical modifiers in the subgroup analyses | The comparison-adjusted funnel plot for the network did not suggest any dominant publication bias | Low (downgrade by two levels due to study limitations and imprecision) |
| **LF-SMA**  **vs**  **LF-OFC** | 100% of the estimate from studies at unclear risk | MD (95% CrI):  0.2 (-7.44 to 7.83) | No head-to-head study and no heterogeneity  Only indirect comparison and no node-splitting inconsistency | The treatment effects were not affected by clinical modifiers in the subgroup analyses | The comparison-adjusted funnel plot for the network did not suggest any dominant publication bias | Low (downgrade by two levels due to study limitations and imprecision) |
| **LF-SMA**  **vs**  **HF-ACC/mPFC** | 50.0% of the estimate from studies at unclear risk, and 50.0% at low risk | MD (95% CrI):  0.08 (-6.25 to 6.34) | No head-to-head study and no heterogeneity  Only indirect comparison and no node-splitting inconsistency | The treatment effects were not affected by clinical modifiers in the subgroup analyses | The comparison-adjusted funnel plot for the network did not suggest any dominant publication bias | Low (downgrade by two levels due to study limitations and imprecision) |
| **LF-OFC**  **vs**  **HF-ACC/mPFC** | 50.0% of the estimate from studies at unclear risk, and 50.0% at low risk | MD (95% CrI):  -0.15 (-8.65 to 8.72) | No head-to-head study and no heterogeneity  Only indirect comparison and no node-splitting inconsistency | The treatment effects were not affected by clinical modifiers in the subgroup analyses | The comparison-adjusted funnel plot for the network did not suggest any dominant publication bias | Low (downgrade by two levels due to study limitations and imprecision) |
| **Ranking**  **of treatment** | 80.9% of the estimate from studies at unclear risk, and 19% at low risk | SUCRA plots suggested the imprecision in a ranking of treatments | Overall I^2^ (73.5%) and P value (< 0.001)  No statistical inconsistency according to the test of global inconsistency (P = 0.442) | The treatment effects were affected by some clinical modifiers in the subgroup analyses | The comparison-adjusted funnel plot for the network did not suggest any dominant publication bias | Very low (downgrade by four levels due to study limitations, imprecision, heterogeneity and indirectness) |

**Table 5 Confidence assessment of drop-out rate by the GRADE system.**

| **Comparison** | **Study limitations** | **Imprecision** | **Heterogeneity and inconsistency** | **Indirectness** | **Publication bias** | **Confidence** |
| --- | --- | --- | --- | --- | --- | --- |
| **Sham**  **vs**  **LF-DLPFC** | 100% of the estimate from studies at unclear risk | OR (95% CrI):  0.75 (0.1, 4.58) | I^2^ (0.0%) and P value (0.990) in direct comparisons  Only direct comparison and no node-splitting inconsistency | The treatment effects were not affected by clinical modifiers in the subgroup analyses | The comparison-adjusted funnel plot for the network did not suggest any dominant publication bias | Low (downgrade by two levels due to study limitations and imprecision) |
| **Sham**  **vs**  **HF-DLPFC** | 100% of the estimate from studies at unclear risk | OR (95% CrI):  1.08 (0.37, 3.19) | I^2^ (0.0%) and P value (1.000) in direct comparisons  Only direct comparison and no node-splitting inconsistency | The treatment effects were not affected by clinical modifiers in the subgroup analyses | The comparison-adjusted funnel plot for the network did not suggest any dominant publication bias | Low (downgrade by two levels due to study limitations and imprecision) |
| **Sham**  **vs**  **LF-SMA** | 100% of the estimate from studies at unclear risk | OR (95%CrI):  0.98 (0.37, 2.67) | I^2^ (0.0%) and P value (0.849) in direct comparisons  Only direct comparison and no node-splitting inconsistency | The treatment effects were not affected by clinical modifiers in the subgroup analyses | The comparison-adjusted funnel plot for the network did not suggest any dominant publication bias | Low (downgrade by two levels due to study limitations and imprecision) |
| **Sham**  **vs**  **LF-OFC** | 100% of the estimate from studies at unclear risk | OR (95% CrI):  0.59 (0.06, 5.68) | I^2^ (0%) and P value (0.804) in direct comparisons  Only direct comparison and no node-splitting inconsistency | The treatment effects were not affected by clinical modifiers in the subgroup analyses | The comparison-adjusted funnel plot for the network did not suggest any dominant publication bias | Low (downgrade by two levels due to study limitations and imprecision) |
| **Sham**  **vs**  **HF-ACC/mPFC** | 100% of the estimate from studies at low risk | OR (95% CrI):  1.62 (0.26, 15.98) | I^2^ (0.0%) and P value (0.545) in direct comparisons  Only direct comparison and no node-splitting inconsistency | The treatment effects were not affected by clinical modifiers in the subgroup analyses | The comparison-adjusted funnel plot for the network did not suggest any dominant publication bias | Moderate (downgrade by one level due to imprecision) |
| **LF-DLPFC**  **vs**  **HF-DLPFC** | 100% of the estimate from studies at unclear risk | OR (95% CrI):  1.44 (0.19, 10.76) | Only one head-to-head study, and no heterogeneity  No inconsistency between the direct and indirect estimate (Node-split p= 0.819) | The treatment effects were not affected by clinical modifiers in the subgroup analyses | The comparison-adjusted funnel plot for the network did not suggest any dominant publication bias | Low (downgrade by two levels due to study limitations and imprecision) |
| **LF-DLPFC**  **vs**  **LF-SMA** | 100% of the estimate from studies at unclear risk | OR (95% CrI):  1.29 (0.18, 13.46) | No head-to-head study and no heterogeneity  Only indirect comparison and no node-splitting inconsistency | The treatment effects were not affected by clinical modifiers in the subgroup analyses | The comparison-adjusted funnel plot for the network did not suggest any dominant publication bias | Low (downgrade by two levels due to study limitations and imprecision) |
| **LF-DLPFC**  **vs**  **LF-OFC** | 100% of the estimate from studies at unclear risk | OR (95% CrI):  0.76 (0.04, 19.87) | No head-to-head study and no heterogeneity  Only indirect comparison and no node-splitting inconsistency | The treatment effects were not affected by clinical modifiers in the subgroup analyses | The comparison-adjusted funnel plot for the network did not suggest any dominant publication bias | Low (downgrade by two levels due to study limitations and imprecision) |
| **LF-DLPFC**  **vs**  **HF-ACC/mPFC** | 55.3% of the estimate from studies at unclear risk, and 44.7% at low risk | OR (95% CrI):  2.24 (0.16, 44.43) | No head-to-head study and no heterogeneity  Only indirect comparison and no node-splitting inconsistency | The treatment effects were not affected by clinical modifiers in the subgroup analyses | The comparison-adjusted funnel plot for the network did not suggest any dominant publication bias | Low (downgrade by two levels due to study limitations and imprecision) |
| **HF-DLPFC**  **vs**  **LF-SMA** | 100% of the estimate from studies at unclear risk | OR (95% CrI):  0.92 (0.22, 4.39) | No head-to-head study and no heterogeneity  Only indirect comparison and no node-splitting inconsistency | The treatment effects were not affected by clinical modifiers in the subgroup analyses | The comparison-adjusted funnel plot for the network did not suggest any dominant publication bias | Low (downgrade by two levels due to study limitations and imprecision) |
| **HF-DLPFC**  **vs**  **LF-OFC** | 100% of the estimate from studies at unclear risk | OR (95% CrI):  0.56 (0.04, 6.81) | No head-to-head study and no heterogeneity  Only indirect comparison and no node-splitting inconsistency | The treatment effects were not affected by clinical modifiers in the subgroup analyses | The comparison-adjusted funnel plot for the network did not suggest any dominant publication bias | Low (downgrade by two levels due to study limitations and imprecision) |
| **HF-DLPFC**  **vs HF-ACC/mPFC** | 51.4% of the estimate from studies at unclear risk, and 48.6% at low risk | OR (95% CrI):  1.49 (0.18, 19.29) | No head-to-head study and no heterogeneity  Only indirect comparison and no node-splitting inconsistency | The treatment effects were not affected by clinical modifiers in the subgroup analyses | The comparison-adjusted funnel plot for the network did not suggest any dominant publication bias | Low (downgrade by two levels due to study limitations and imprecision) |
| **LF-SMA**  **vs**  **LF-OFC** | 100% of the estimate from studies at unclear risk | OR (95% CrI):  0.61 (0.05, 6.5) | No head-to-head study and no heterogeneity  Only indirect comparison and no node-splitting inconsistency | The treatment effects were not affected by clinical modifiers in the subgroup analyses | The comparison-adjusted funnel plot for the network did not suggest any dominant publication bias | Low (downgrade by two levels due to study limitations and imprecision) |
| **LF-SMA**  **vs HF-ACC/mPFC** | 50.0% of the estimate from studies at unclear risk, and 50.0% at low risk | OR (95% CrI):  1.64 (0.22, 20.28) | No head-to-head study and no heterogeneity  Only indirect comparison and no node-splitting inconsistency | The treatment effects were not affected by clinical modifiers in the subgroup analyses | The comparison-adjusted funnel plot for the network did not suggest any dominant publication bias | Low (downgrade by two levels due to study limitations and imprecision) |
| **LF-OFC**  **vs HF-ACC/mPFC** | 50.0% of the estimate from studies at unclear risk, and 50.0% at low risk | OR (95% CrI):  2.96 (0.15, 61.58) | No head-to-head study and no heterogeneity  Only indirect comparison and no node-splitting inconsistency | The treatment effects were not affected by clinical modifiers in the subgroup analyses | The comparison-adjusted funnel plot for the network did not suggest any dominant publication bias | Low (downgrade by two levels due to study limitations and imprecision) |
| **Ranking**  **of treatment** | 80.6% of the estimate from studies at unclear risk, and 19.3% at low risk | SUCRA plots suggested the imprecision in a ranking of treatments | Overall I^2^ (0.0%) and P value (1.000)  No statistical inconsistency according to the test of global inconsistency (P = 0.987) | The treatment effects were not significantly affected by clinical modifiers in the subgroup analyses | The comparison-adjusted funnel plot for the network did not suggest any dominant publication bias | Low (downgrade by two levels due to study limitations and imprecision) |
